# Towards Sustainable Bioleaching of Platinum Group Metals from Spent Automotive Catalysts

**DOI:** 10.3390/ma19163495

**Published:** 2026-08-18

**Authors:** Yeskalina Kuralay, Zahra Ilkhani, John Hardy, Luigi Capozzi, Farid Aiouache

**Affiliations:** 1 Department of Metallurgy and Mineral Processing, Satbayev University, Almaty 050013, Kazakhstan; k.yeskalina@lancaster.ac.uk; 2 Department of Metallurgy and Mining, K. Zhubanov Aktobe Regional University, Aktobe 030000, Kazakhstan; 3 School of Engineering, Lancaster University, Lancaster LA1 4YR, UK; z.ilkhani@lancaster.ac.uk (Z.I.); j.g.hardy@lancaster.ac.uk (J.H.); l.capozzi@lancaster.ac.uk (L.C.)

**Keywords:** bioleaching, platinum group metals, spent automotive catalysts, cyanogenic bacteria, *Acidithiobacillus*, shrinking core model

## Abstract

Spent automotive catalysts represent an important secondary resource for platinum group metals, offering environmental and economic advantages over primary mining. This review evaluates bioleaching-based recovery strategies of these metals as sustainable alternatives to conventional pyrometallurgical and hydrometallurgical processing. The cyanogenic bioleaching using *Chromobacterium violaceum*, *Pseudomonas fluorescens*, and *Bacillus megaterium*, and acidophilic bioleaching using *Acidithiobacillus* spp. for washcoat degradation and base-metal removal are discussed through the one-step, two-step, spent-medium, and decoupled systems. The analysis shows progressive improvement of recovery as process separation increases. Sequential pretreatment involving ultrasound-assisted acid leaching, thermal oxidation, and pressure-enhanced processing improved recovery by removing competing base metals and increasing PGM accessibility. Kinetic analyses indicate that diffusion through the porous catalyst support matrix becomes the dominant rate-controlling mechanism at high conversion, which impacts reactor design. Despite sustainability potential, industrial implementation remains constrained by low pulp density, cyanide stability, reactor productivity, and scale-up limitations. Routes to commercialisation require feasibility studies of process designs that integrate viable process flow diagrams combining pretreatment, biological lixiviant generation, intensified bioleaching, and downstream metal purification.

## 1. Introduction

Platinum group metals (PGMs), known by the three species of Pt, Pd, and Rh, used as main catalysts in automotive catalytic converters to reduce NO_x_, CO, and hydrocarbon emissions from engines, account for more than 56% of PGMs consumed over the past decade and amount to about 565 tons in 2023 [1,2]. Present in spent automotive catalysts (SACs) at about 0.1–0.3 wt.%, they are more available than in primary ores at about 2–10 ppm [3,4] and exhibit less environmental impact, e.g., consuming 70–100 times less energy compared to mining [5], and position them as one of the most important secondary sources for sustainable PGM recovery [6].

Conventional SAC recycling primarily relies on pyrometallurgical smelting and hydrometallurgical leaching with strong acids or cyanide salts and is considered technically satisfactory for effective PGM removal. These approaches can achieve high PGM recovery yields, however, from an environmental perspective, conventional SAC releases significant amounts of emissions and hazardous chemicals, necessitating complex purification steps [7]. Bioleaching, as a sustainable, energy-saving option that uses microbial metabolism, has attracted attention compared to conventional methods such as pyrometallurgy and hydrometallurgy (Table 1). Pyrometallurgy is currently an industrially mature technology, as it offers high throughput and the recovery of Pt, Pd, and Rh, but it requires high operating temperatures (typically above 1000 °C, often around 1450–1600 °C depending on the smelting route) and high energy consumption, resulting in significant CO_2_ emissions and environmental burdens. Hydrometallurgy operates at moderate conditions and provides improved selectivity for PGMs but relies on aggressive chemical lixiviants, such as strong acids and cyanide salts, which increase waste treatment requirements and secondary environmental impacts. In contrast, biohydrometallurgy operates at mild temperatures, using microorganisms to generate lixiviants in situ while reducing chemical consumption and energy demand. Although the bioleaching method remains insufficiently understood in terms of its chemical kinetics and sensitivity to process conditions, it demonstrates sustainability potential and can achieve high recovery efficiencies when integrated with suitable pretreatment strategies. Two main biological approaches for recovering PGMs have been studied. The first is cyanogenic bioleaching, which uses bacteria such as *C. violaceum*, *P. fluorescens*, and *B. megaterium* to produce hydrogen cyanide by breaking down glycine. It operates in alkaline conditions, where cyanide ions form stable, soluble complexes with Pt, Pd, and Rh, allowing these metals to dissolve selectively from SACs [7,8,9]. The second is acidophilic bioleaching, which uses bacteria such as *Acidithiobacillus* species to produce sulfuric acid and ferric iron, which break down alumina washcoats and base metals, making PGMs more accessible for subsequent recovery steps [10].

More recent research often combines these methods in integrated or step-by-step processes. Acidophilic pretreatment is usually used first to remove base metals and partly break down catalyst materials before cyanogenic leaching. This approach can reduce the amount of lixiviant required and makes the metals easier to recover [10,16,17]. Other improvements, such as using ultrasound with acid pretreatment, pressure bio-cyanidation, reducing oxidative stress, and extracting with ionic liquids, have been reported to increase PGM recovery rates when conditions are optimized [18,19].

This review examines current progress in PGM bioleaching from SACs, focusing on sequential pretreatment methods, cyanogenic and acidophilic processes, microbial performance, process integration, leaching kinetics, sustainability, and economic factors. Special attention is given to the transition from lab-scale experiments to practical industrial bio-hydrometallurgical processes. The literature for this review was identified through comprehensive searches of major scientific databases, including Scopus, Web of Science, and Google Scholar, using combinations of keywords related to spent automotive catalysts, PGM, bioleaching, biocyanidation, leaching, and critical metal recovery. More than 250 publications on PGM recovery from spent automotive catalysts were initially screened, and those focusing specifically on bioleaching of base metals and biocyanidation were then selected. Following the removal of duplicate records and the classification of publications according to their relevance, originality, and scientific contribution to the scope of this review, the publications referenced herein were retained. Priority was given to peer-reviewed articles that provided significant advances in biological recovery mechanisms, process optimisation, engineering aspects, and sustainability assessment of PGM recovery from spent automotive catalysts.

## 2. Pretreatment Strategies

The direct bioleaching of PGMs from SACs is hindered by four interconnected barriers: physical, chemical, mass transfer, and biological. Physical barriers mainly arise from particle encapsulation and restricted surface accessibility, both of which reduce lixiviant contact with embedded PGMs. The chemical barriers could arise from alumina washcoats, stable oxide phases, and competing base metals that limit metal mobilisation and increase reagent consumption. Biological barriers include microbial sensitivity to toxic metals and environmental conditions, as well as diffusion resistance, which can limit reagent penetration and create mass-transfer limitations that slow overall extraction kinetics. Without pretreatment, solubilization of Pt from the solid catalyst matrix is notoriously low, sometimes yielding only 0.2% platinum extraction [20].

To overcome these independent barriers, various pretreatment strategies have been developed to disrupt the physical encapsulation, eliminate chemical passivation, enhance mass transfer, and improve microbial activity prior to the main leaching step. Recovering PGMs from SACs is challenging since PGMs are dispersed throughout alumina-rich washcoats and cordierite supports, chemically stabilised, and often covered by carbonaceous deposits generated during catalyst operation [21,22]. SACs also contain several non-PGM elements, including Fe, Ni, Al, Ti, Zn, Cu, Ba, and Zr, as well as rare-earth elements such as Ce and La, along with the target precious metals Pt, Pd, and Rh [7,18,23]. These base metals, particularly Cu and Zn, can compete with PGMs for lixiviants such as cyanide, therefore increasing reagent consumption, and potentially inhibiting microbial activity during bioleaching. Consequently, pretreatment is generally required to improve catalyst accessibility and minimise competing reactions [18].

Ultrasound-assisted acid leaching has been demonstrated to be effective for removing interfering base metals. Karim and Ting [9] reported that optimised sonochemical acid treatment removed approximately 80% of Cu and Zn through particle fragmentation, surface erosion, and enhanced mass transfer. Because Cu and Zn readily form cyanide complexes, their removal reduces cyanide consumption and improves selective PGM dissolution (Equations (1) and (2)) [24,25]. The quantitative effects of ultrasound-assisted pretreatment are summarised in Table 2.

(1) $$Cu^{+}+2CN^{-}⇌[Cu(CN{)}_{2}{]}^{-}\;\log\beta =\sim 27$$

(2) $$Zn^{2+}+4CN^{-}⇌[Zn(CN{)}_{4}{]}^{2-}\;\log\beta =\sim 19$$

Thermal oxidation and reductive passivation conditioning steps are also relevant to overcome surface passivation by removing carbonaceous deposits, modifying surface oxide phases using formic or ascorbic acid and restoring metallic surface reactivity prior to cyanogenic leaching. The combination of thermal oxidation, ultrasound-assisted acid leaching, and reductive activation has been shown to increase recoveries to 91% Pt, 95% Pd, and nearly 100% Rh when coupled with engineered *C. violaceum* systems [8].

Biological pretreatment can condition catalyst matrices through selective dissolution of alumina phases and competing base metals, thereby increasing catalyst porosity and improving accessibility of encapsulated PGMs [10]. Similar improvements have been reported using moderately thermophilic sulfur-oxidising consortia and fungi such as *Aspergillus niger* [26,27]. These microorganisms primarily condition the catalyst matrix rather than directly dissolve PGMs. The mechanisms and performance of biological pretreatment systems are discussed further in Section 4 and Section 5. Table 2 illustrates the determinant role of pretreatment on leaching performance. Individual pretreatment methods yielded improvements, but the highest leaching rates were generally achieved with integrated strategies combining thermal, mechanical, chemical, and biological techniques. Such approaches improve lixiviant accessibility, reduce reagent consumption, and enhance dissolution kinetics during subsequent bioleaching.

## 3. Cyanogenic Bacterial Strains

The primary difference in how autotrophic and heterotrophic microorganisms leach metals lies in their energy sources and metabolic activities. Autotrophic bacteria, commonly known as sulfur-oxidizing and iron-oxidizing bacteria, which are capable of growing in acidic conditions, are the most prevalent. During the bioleaching process, these microorganisms facilitate the oxidation of ferrous ions to ferric ions and elemental sulfur to sulfuric acid.

Cyanogenic bioleaching remains the most extensively investigated biological approach for recovering PGMs from SACs as cyanide forms highly stable aqueous complexes with Pt, Pd, and Rh under alkaline conditions. However, effective PGM dissolution depends not only on cyanide production but also on microbial tolerance to dissolved metals, metabolic robustness, and compatibility with bioleaching conditions. Among these microorganisms, *C. violaceum* has been the most widely studied, as it has consistently demonstrated the highest bioleaching performance. Cyanide is produced through the oxidative decarboxylation of glycine by the membrane-bound HCN synthase complex encoded by the *hcnABC* operon Equation (3):

C_2_ H_5_ NO_2_ + O_2_ →HCN + CO_2_ + 2H_2_O (3)

**Figure 1:**
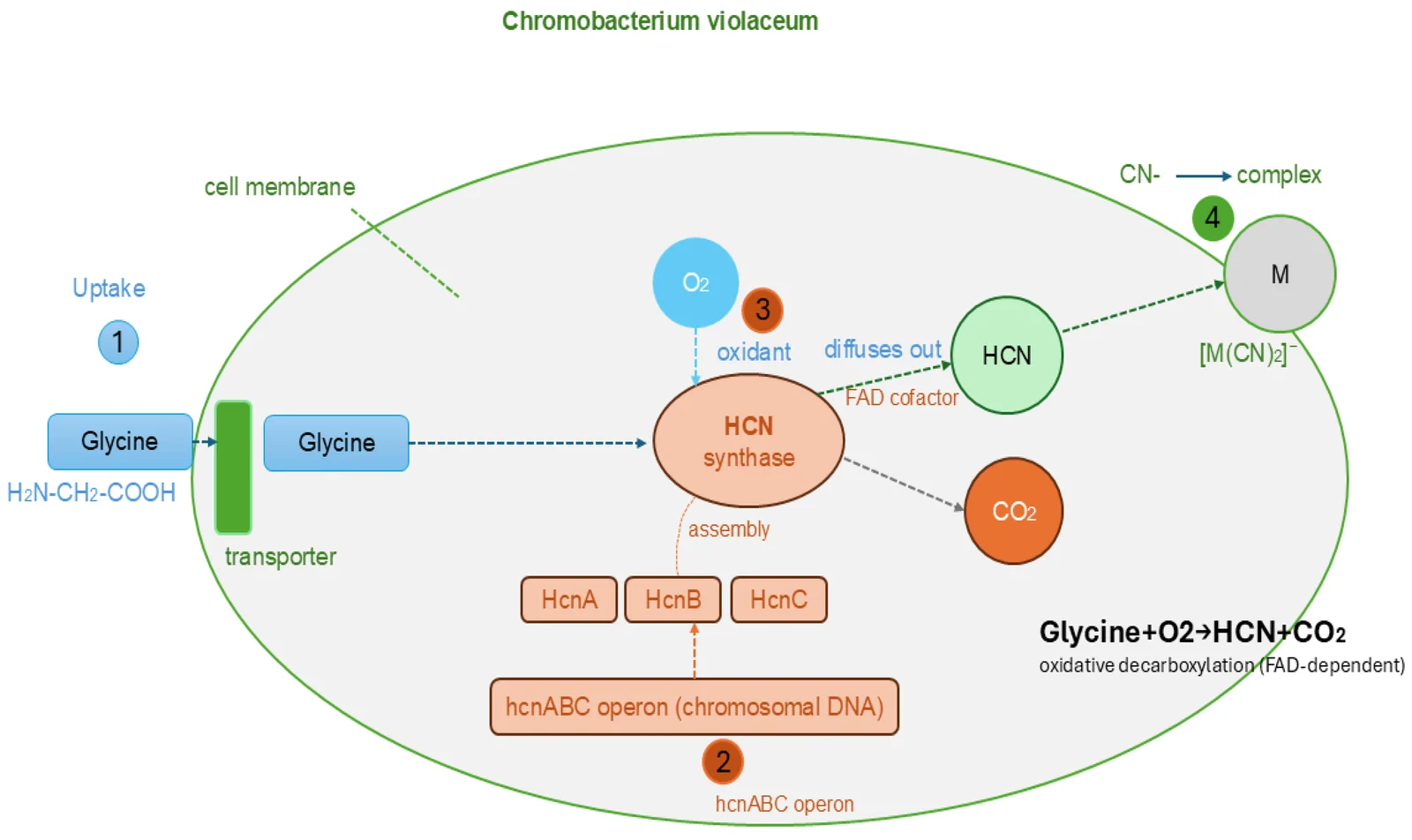
Biochemical pathway of HCN production in *C. violaceum*. Numbers 1–4 indicate the sequential steps of glycine uptake, hcnABC expression and HCN synthase assembly, HCN production, and PGM–cyanide complex formation, respectively. Arrows indicate the direction of transport and biochemical reactions. Blue colour: substrates/reactants and uptake; orange colour: HCN synthase and reaction products; green colour: cellular components and PGM complexation.

Cyanide production is influenced by glycine availability, oxygen transfer, cellular metabolism, and growth phase, with maximum production generally occurring during the transition from exponential to stationary growth phase [30]. The biochemical pathway of cyanide generation and its role in PGM complexation are illustrated in Figure 1. As shown, *C. violaceum* takes up glycine via a membrane transporter (step 1), the *hcnABC* operon is transcribed and translated to assemble HCN synthase from *HcnA*, *HcnB*, and *HcnC* subunits (step 2), and HCN synthase catalyses the oxidative decarboxylation of glycine using O_2_ to yield HCN and CO_2_ (step 3). The HCN then diffuses out of the cell and forms water-soluble complexes with PGMs (step 4) such as [Pt(CN)_4_]^2−^, [Pd(CN)_4_]^2−^, and [Rh(CN)_6_]^3−^, enabling metal dissolution into solution [9,28,30,31,32].

The bioleaching process is carried out through direct and indirect methods. The direct method is classified into the one-step and two-step methods, while the indirect method involves cyanide production in *C. violaceum* and other cyanogenic bacteria and is broadly governed by both metabolic state and precursor supply. Mechanistic studies in *Pseudomonas aeruginosa*, which shares the same core biosynthetic pathway, have provided much of the foundational understanding. Castric [33] demonstrated that HCN is a secondary metabolite whose formation begins as growth rate declines and is influenced by oxygen availability, iron, phosphate, temperature, and protein synthesis. In another study, glycine was identified as the principal precursor of HCN, while threonine and serine contributed indirectly through conversion to glycine; methionine enhanced cyanogenesis without serving as an effective carbon precursor [34]. Taken together with the broader microbial cyanogenesis literature, these findings indicate that HCN biosynthesis is linked to late-exponential and subsequent idiophase metabolism and likely functions in the regulation of excess glycine and related amino acid pools [30,33,34].

Cyanide concentrations obtained in batch cultivation typically remain below several hundred mg L^−1^, which limits dissolution rates. Recent research has therefore focused on metabolic engineering and process intensification. Ilyas et al. [32] further demonstrated that *C. violaceum* under continuous and pressurised operation can reach cyanide concentrations of up to 4170 mg L^−1^, achieving Pt and Pd dissolution efficiencies exceeding 95% under leaching conditions of 150 °C and 14 bar O_2_ over 120 min, with subsequent ionic-liquid extraction yielding recoveries above 96% for both metals. *P. plecoglossicida*, another example of a cyanogenic species, has shown limited success in SAC processing. Brandl et al. [20] reported that the bacteria produced less than 5 mg L^−1^ of cyanide, which mobilised only 0.2% of Pt from spent automotive catalytic converters after 10 days, an effect attributed to the formation of a passivating oxide film on the Pt surface.

## 4. Acidophilic Chemolithotrophs

Alongside cyanogenic microorganisms, acidophilic chemolithotrophs are emerging as important matrix-conditioning agents for SAC processing. The acidophilic microorganisms facilitate indirect recovery by generating acidic and oxidising environments that remove alumina washcoats and competing base metals such as Fe, Cu, Ni, and Zn [12,35,36]. These actions improve access to encapsulated PGMs while reducing cyanide consumption and metal toxicity during subsequent cyanogenic leaching.

The acid-bioleaching pretreatment of SACs using acidophilic species represents a promising green chemistry approach for disrupting the refractory α-alumina washcoat that encapsulates PGMs, including Pt, Pd, and Rh. Compagnone et al. [10] demonstrated this using *Acidithiobacillus thiooxidans* to generate biogenic sulfuric acid, achieving 54.5% aluminium dissolution at a pulp density of 5% (*w*/*v*). Notably, the biogenic acid produced by stationary-phase cells was significantly more effective than both commercial H_2_SO_4_ (24.7% Al extraction) and cell-free biogenic acid (23.4% Al) with exponential-phase bacteria (21.7%), highlighting the importance of active microbial metabolism and associated microenvironmental effects between the exponential and stationary phases and role of adaptation to stress factors by spontaneous activation of survival mechanisms in the former to update their gene expression patterns. Furthermore, the dissolution process induced the production of extracellular polymeric substances (EPS), which adsorbed a fraction of the solubilised aluminium (up to 24.8%) and trace amounts of Pt (up to 0.40%), thereby introducing an additional constraint on downstream metal recovery. These limitations were earlier partially overcome by the approach reported by Pakostova and Rowson [17], who demonstrated that an indirect bioleaching strategy employing a mixed consortium of moderately thermophilic acidophiles at 50 °C can dissolve over 90% of the alumina-rich matrix. The synergistic activity of *Acidithiobacillus caldus*, *Sulfobacillus thermosulfidooxidans*, *Sulfobacillus acidophilus*, and *Alicyclobacillus siderooxidans* enhances sulfur and iron oxidation rates, leading to sustained acid generation and improved leaching kinetics.

Beyond SACs, acidophilic bioleaching has been applied across a broad range of PGM-bearing and metal-bearing substrates, illustrating both the mechanistic versatility of *Acidithiobacillus* dominated systems and their potential as a general matrix-conditioning platform. *A. ferrooxidans* has been characterised as the benchmark organism for acidic bioleaching, with genome-based studies elucidating the sulfur and iron oxidation pathways that provide energy and underpin industrial-scale sulfide mineral dissolution with reduced power requirements [35]. Representative acidophilic bioleaching across different substrate types is summarised in Table 3.

Although acidophilic systems generally exhibit slower kinetics and limited direct dissolution of Pt, Pd, and Rh compared with cyanogenic bioleaching, they offer several advantages, including in situ acid generation, reduced chemical consumption, tolerance to elevated metal concentrations, and suitability for large-scale matrix conditioning. These properties have led to their increasing use as the first stage of hybrid bio-hydrometallurgical flowsheets, where they prepare catalyst materials for more selective downstream cyanogenic recovery.

## 5. Mechanisms of Bacteria in PGM Liberation

### 5.1. Cyanogenic Mechanisms and PGM Complexation

**Figure 2:**
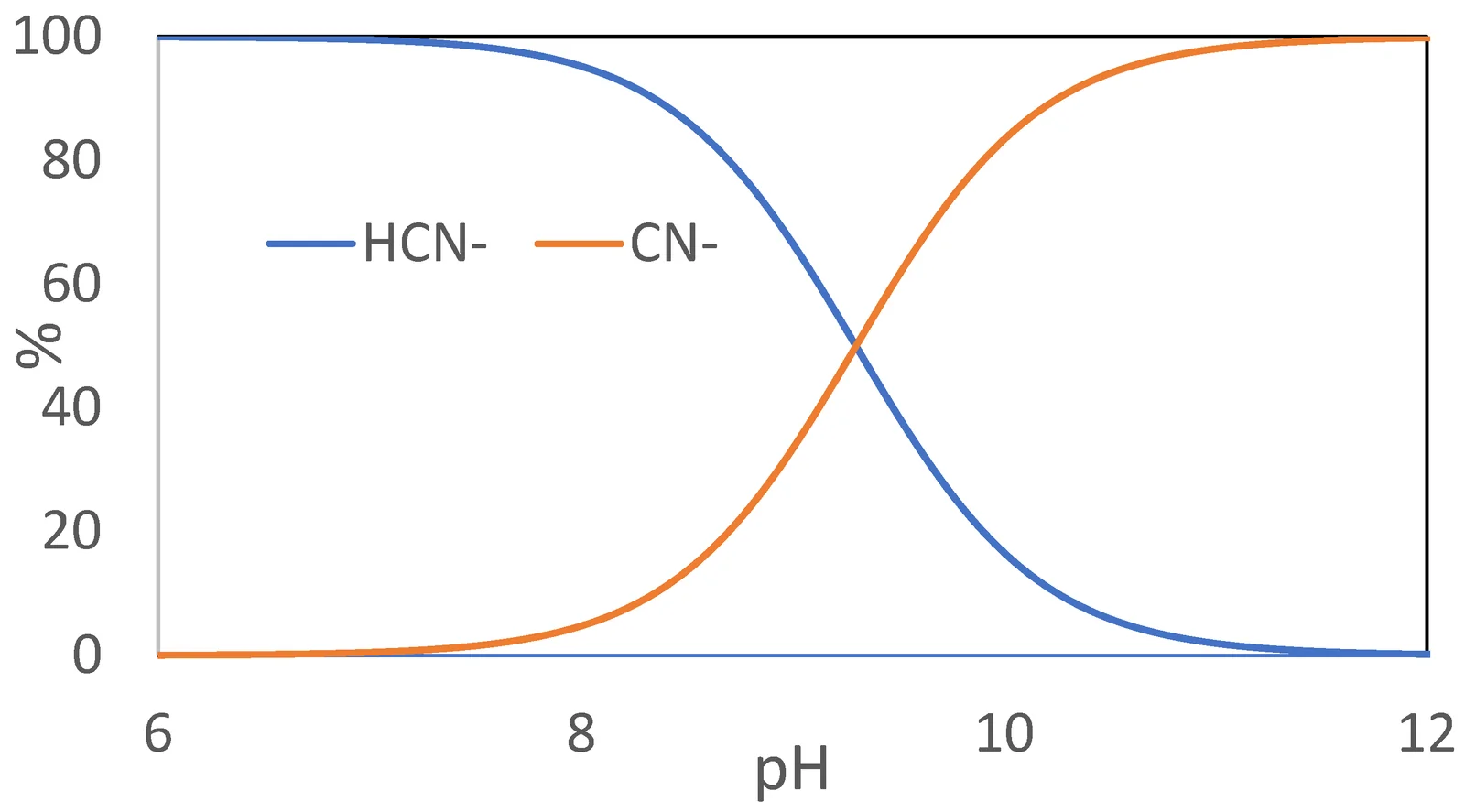
Influence of pH on cyanide speciation (25 °C, pKa of HCN ≈ 9.3). According to the Henderson-Hasselbalch model.

According to the acid dissociation constant (pKa ≈ 9.3 at 25 °C) of hydrogen cyanide, cyanide exists mainly as dissolved CN^−^ anions under alkaline medium. Maintaining pH values between approximately 9 and 11 therefore minimises HCN volatilisation and maximises cyanide availability for metal complexation. The influence of pH on cyanide speciation is illustrated in Figure 2 [30,41]. PGM dissolution proceeds via oxidative cyanidation, producing stable, soluble cyanometallate complexes. The reactions for PGM dissolution are shown in Equations (4) and (5) [42].

(4) $$Pt+4CN^{-}+O_{2}+2H_{2}O\to [Pt(CN{)}_{4}{]}^{2-}+4OH^{-}$$

(5) $$Pd+4CN^{-}+O_{2}+2H_{2}O\to [Pd(CN{)}_{4}{]}^{2-}+4OH^{-}$$

The effectiveness of cyanidation reactions depends not only on the presence of cyanide but also on catalyst properties, including morphology, oxygen transfer, and accessibility of the active metal surface. Surface passivation by PGM oxides, such as PtOx, PdO, and Rh_2_O_3_, can significantly reduce dissolution rates by limiting electron transfer and ligand interactions at the metal interface [13,43]. Reductions by agents such as formic acid or ascorbic acid help restore the reactivity of metallic surfaces [8]. There is a challenge that arises from the differing pH requirements of microbial growth and metal dissolution. The cyanogenic bacteria, such as *C. violaceum*, *P. fluorescens*, and *B. megaterium* generally exhibit optimum growth near neutral pH, whereas efficient cyanide complexation requires alkaline conditions [8,14,44]. Excessively alkaline environments can therefore impair cellular metabolism by disrupting membrane-associated proton gradients and enzyme activity. Consequently, the conditions that favour cyanide production are not necessarily identical to those that maximise metal dissolution.

A further limitation arises from competing base metals present within SAC matrices. Metals such as Cu, Ni, Zn, and Fe form stable cyanide complexes and therefore consume significant quantities of available cyanide. Cu and Zn, for instance, have a high affinity for CN^−^ as illustrated by Equations (1) and (2). Because these metals are commonly present at concentrations substantially higher than those of PGMs, they decrease selectivity and increase lixiviant demand. Relative cyanide affinities and complex stabilities are summarised in Table 4. Among the competing metals that contribute to cyanide losses, Cu and Ni exhibit strong cyanide affinities, whereas ferric iron forms ferricyanide. Pt and Pd retain sufficiently high stability constants (log β ≈ 40–43) to form highly stable cyanometallate complexes. Rh exhibits similar thermodynamic behaviour, but its dissolution kinetics are slower. Overall, successful cyanogenic bioleaching depends on keeping favourable cyanide speciation, minimising surface passivation, and limiting competitive cyanide consumption by coexisting metals. These factors collectively govern lixiviant efficiency and ultimately determine PGM recovery.

### 5.2. Acidophilic Mechanisms and Matrix Degradation

Bioleaching that relies on acidophilic bacteria primarily follows three complementary mechanisms: (i) the acidolysis via biogenic sulfuric acid production. (ii) the ferric-iron-mediated redoxolysis, and (iii) the interfacial dissolution that is enhanced by microbial biofilms and extracellular polymeric substances (EPS). During sulfur oxidation, *A. thiooxidans* convert elemental sulfur into sulfuric acid (Equation (6)), which promotes dissolution of alumina-rich washcoats and associated oxide phases (Equation (7)) [49].

(6) $$S^{0}+1.5O_{2}+H_{2}O\to H_{2}SO_{4}$$

(7) $$Al_{2}O_{3}+6H^{+}\to 2Al^{3+}+3H_{2}O$$

In practice, the dissolution of γ- and θ-alumina phases typically proceeds more readily than α-alumina due to their higher surface area, structural disorder, and lower thermodynamic stability [10]. Under the mild acidic conditions typical of acidophilic bioleaching, α-Al_2_O_3_ is considerably more chemically refractory than γ-Al_2_O_3_, consistent with its lower surface area and greater structural order. While prolonged exposure to more aggressive acidic conditions or elevated temperatures could, in principle, promote further attack on the α-phase, such conditions are not typically employed in acidophilic bioleaching due to their inhibitory effects on microbial activity. In practice, the benefits of acidophilic pretreatment on PGM accessibility are therefore considered to arise primarily from dissolution of the more reactive γ-Al_2_O_3_ washcoat phases, together with base-metal removal and physical disruption of the matrix, rather than from extensive α-Al_2_O_3_ dissolution [10].

As γ-Al_2_O_3_ which is also called as transition alumina, is the metastable forms of aluminum oxide, while another common alumina polymorph, α-Al_2_O_3_, is much more stable and usually crystallized at high temperatures. if γ-Al_2_O_3_ can be transformed into α-Al_2_O_3_, the leaching of Al is expected to decrease. It is helpful to simplify the subsequent Al-separation process and improve the overall recovery efficiency of valuable metals. Moreover, the final leaching residues with high α-Al_2_O_3_ purity can be utilized to prepare high value-added materials [50].

The main acidophilic mechanism enhancing metal leaching is the continuous production of biogenic sulfuric acid by *A. thiooxidans* through the oxidation of elemental sulfur (Equation (6)). The synergistic effect of the biogenic sulfuric acid, the highly acidic environment (pH ~ 1), and the presence of metabolic activity of bacteria results in high dissolution of the alumina layer. In a study by Compagnone et al. [10], microscopic observations further revealed that *A. thiooxidans* cells attached directly to the catalyst surface, enabling sulfuric acid to be generated at the mineral-solution interface rather than only in the bulk solution. This acid production accelerated the dissolution of the α-alumina washcoat at the solid-liquid interface, thereby enhancing the removal of the catalyst matrix [10,51].

A second important mechanism involves the generation of ferric iron. *A. ferrooxidans* catalyses the oxidation of ferrous iron according to Equation (8):

(8) $$2Fe^{2+}+\frac{1}{2}O_{2}+2H^{+}\to 2Fe^{3+}+H_{2}O$$

The resulting ferric iron acts as a strong oxidant, capable of dissolving iron-bearing phases and promoting the breakdown of associated base-metal compounds (e.g., Fe, Ni or Cu containing phases). This process not only contributes to structural weakening of the catalyst matrix but also removes competing metals that would otherwise consume lixiviants during downstream PGM recovery. In addition, ferric iron can undergo cyclic regeneration via microbial oxidation, sustaining redox activity in the system.

Microbial attachment to the catalyst surface further enhances bioleaching through biofilm formation and EPS secretion. These EPS create a localised microenvironment at the mineral microbe interface that differs significantly from the bulk solution in terms of pH, ionic strength, and redox potential. In sulfur-oxidising systems dominated by *A. thiooxidans*, EPS retains high concentrations of protons (H^+^) and sulfate (SO_4_^2−^) near the solid surface, thereby intensifying proton-promoted dissolution of alumina. This interfacial process, often described as the contact mechanism, can lead to higher apparent dissolution rates than those predicted by bulk diffusion alone. In contrast, non-contact mechanisms involve lixiviant production in solution followed by diffusion to the solid surface; in practice, both pathways operate simultaneously, with their relative contributions governed by pulp density, mixing conditions, and biofilm development.

In SAC systems, EPS also play a dual physicochemical role. In addition to facilitating interfacial acid attack, they adsorb solubilised aluminium and trace PGM species from the leachate via functional groups such as carboxyl, hydroxyl, and phosphoryl moieties. Compagnone et al. [10] reported that up to dissolved Al and Pt were retained within the EPS fraction, indicating that biosorption proceeds concurrently with acidolysis. This introduces a competing effect: while biosorption may temporarily reduce dissolved metal concentrations and apparent leaching efficiency, it also localises metals within the biomass phase, enabling potential recovery through desorption, biomass treatment, or thermal processing.

It should be noted that pH influences the availability of the competing base metals such as Fe, Al, Cu, and Zn. These metals remain relatively soluble in acidic conditions and contribute to metal toxicity, lixiviant consumption, and secondary mineral formation. Under alkaline conditions, however, many of these species precipitate as hydroxides, thereby reducing dissolved metal concentrations but potentially forming surface coatings that restrict lixiviant access. pH affects not only PGM dissolution but also impurity behaviour and overall process selectivity. The interaction between pH and temperature further complicates the monitoring of the process. High temperatures would accelerate PGM dissolution kinetics but may also increase cyanide degradation rates [52,53]. However, above 160 °C, the leaching efficiency decreases due to reduced oxygen availability and promoted cyanide hydrolysis. Similarly, temperature influences microbial activity, sulfur oxidation, and ferric iron generation in acidophilic systems. Effective process design, therefore, requires the simultaneous optimisation of pH, temperature, oxygen availability, pulp density, and microbial physiology rather than considering any single parameter in isolation.

Importantly, despite the significant contribution of these mechanisms to alumina dissolution and increased PGM accessibility, the direct bioleaching of Pt, Pd, and Rh remains negligible under acidic, non-complexing conditions. These noble metals are thermodynamically stable and require strong complexing ligands (e.g., CN^−^, S_2_O_3_^2^^−^, or halides) under oxidising conditions to form soluble species.

### 5.3. Kinetics of PGM Leaching

#### 5.3.1. Mass-Transfer Constraints

**Figure 3:**
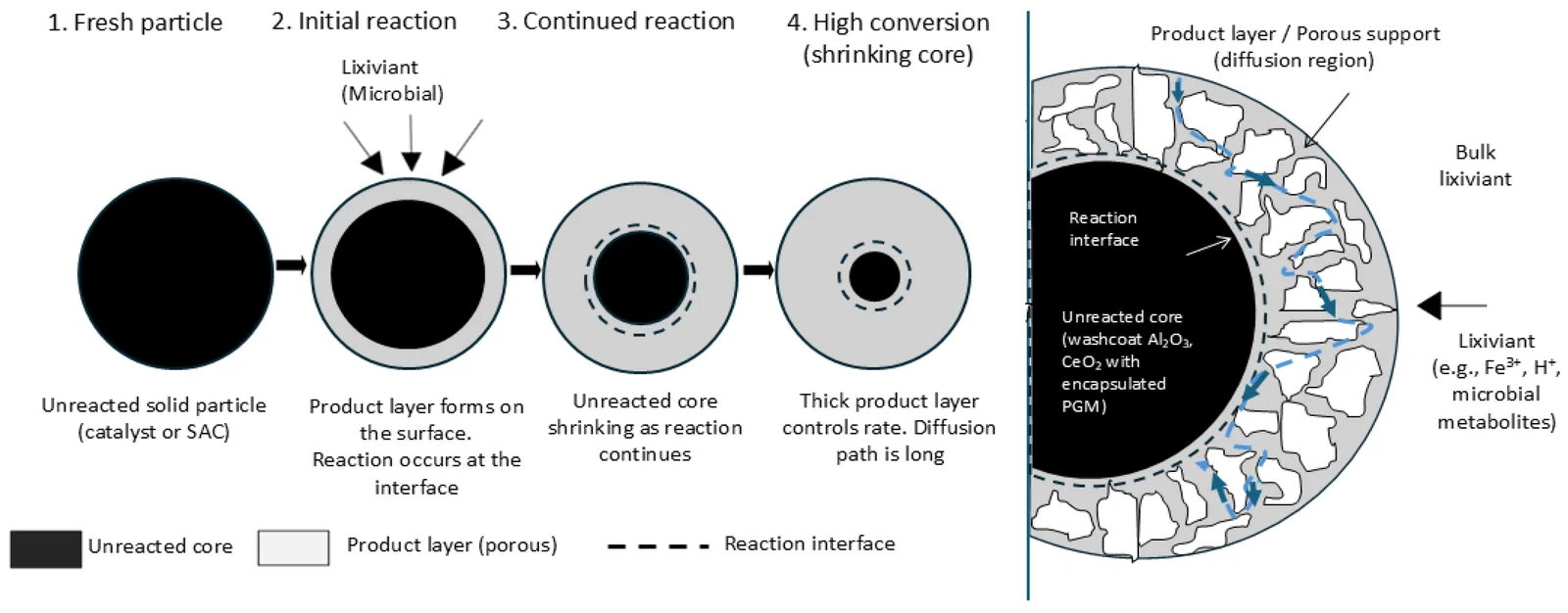
Diffusion-controlled shrinking-core mechanism in SACs bioleaching [54,55].

Despite the effectiveness of biologically generated lixiviants, PGM bioleaching remains limited by relatively slow dissolution rates. At high conversion levels, diffusion through porous catalyst structures and reaction-product layers often becomes the dominant rate-limiting step. Consequently, shrinking-core-type models (SCM) frequently provide a better description of SAC bioleaching behaviour than purely surface-reaction-controlled models. The conceptual framework of SCM is illustrated in Figure 3.

Initially, the biogenic lixiviants would readily react with accessible PGM particles near the catalyst surface. As the leaching process progresses, dissolution increasingly depends on reagent mass transport through porous alumina washcoats, cordierite supports, and reaction-product interface layers. This transition from reaction-controlled to diffusion-controlled behaviour would explain the progressive reduction in leaching rates observed at high conversion.

It should be noted that several process intensification strategies have been proposed to improve mass transfer rate and lixiviant availability. Enhanced slurry mixing, continuous cyanide generation, reactor decoupling, and pressure cyanidation have all been reported to improve dissolution performance. For example, continuous cyanide-generation systems coupled with pressure cyanidation achieved dissolution efficiencies exceeding 90% for Pt, Pd, and Rh [28]. These observations suggest that future improvements in PGM bioleaching will increasingly depend on overcoming diffusion limitations within catalyst matrices rather than solely on increasing microbial activity.

#### 5.3.2. Rate-Determining Steps and Modelling

Unlike the conventional base-metal bioleaching systems, PGM dissolution from SACs involves coupled biological, chemical, and transport phenomena. Most studies report an initial period of relatively rapid metal dissolution, followed by a slower stage controlled primarily by diffusion through porous catalyst structures and reaction products [54,56]. Within the shrinking-core model, overall leaching rates may be controlled by external film diffusion (Equation (9)), surface chemical reaction (Equation (10)), or diffusion through a porous product layer (Equation (11)).

(9) $$F=k_{1}t$$

(10) $$1-(1-F{)}^{\frac{1}{3}}=k_{2}t\;$$

(11) $$1-\frac{2}{3}F-(1-F{)}^{2/3}=k_{3}t$$

where *F* represents fractional conversion and *k*_1_–*k*_3_ are apparent rate constants [54]. Most SAC bioleaching studies indicate that product-layer diffusion becomes dominant at high conversion [27,54,55,56]. However, classical SCM assumptions should be applied cautiously because microbial activity continuously generates lixiviants, violating the pseudo-steady-state conditions assumed in conventional models.

At low pulp densities, the early-stage cyanogenic dissolution is often described by pseudo-first-order kinetics (Equation (12)).

(12) ln(CtC0)=−kt

where *C*_0_ and *C*_t_ represent the concentrations of PGM remaining in the solid phase at the initial time and at time t, respectively. Several studies have additionally proposed Langmuir-Hinshelwood-type behaviour, suggesting that cyanide and oxygen adsorb onto metal surfaces prior to reaction (Equation (13)).

(13) r=kKC1+KC

where *r* is the reaction rate, *k* is the rate constant, *K* is the equilibrium adsorption constant, and *C* is the cyanide concentration. This model explains why dissolution rates may plateau beyond an optimum cyanide concentration due to competitive adsorption and cyanide decomposition reactions. High temperatures and oxygen pressures accelerate dissolution kinetics of Pt and Pd by increasing reaction rates and oxygen availability. Under optimised pressure conditions, biogenic cyanide has achieved recoveries comparable to those obtained using chemical cyanide solutions [28,57].

From a biological perspective, cyanide production is often described using modified Monod-type growth kinetics (Equation (14)).

(14) $$r=\frac{\mu_{max}S}{K_{s}+S}$$

where *μ* represents the specific growth rate, *μ*_max_ is the maximum growth rate, *S* is substrate concentration, and *K*_s_ is the half-saturation constant. Because significant cyanide production typically occurs during late exponential growth, an initial lag period commonly precedes substantial PGM dissolution.

Overall, current evidence indicates that PGM bioleaching cannot be adequately described by a single kinetic mechanism. Instead, dissolution should be viewed as a coupled multi-scale process involving microbial growth, lixiviant generation, interfacial reactions, and intra-particle diffusion. Hybrid kinetic models integrating microbial and transport phenomena are therefore likely to provide the most realistic basis for reactor design and scale-up.

## 6. Bioleaching Strategies Analysis

The choice of bioleaching configuration strongly influences PGM recovery because microbial growth, lixiviant generation, lixiviant stability, and metal dissolution require different operating conditions. Recovery performance generally follows the trend:

one-step<two-step<spent-medium<decoupled systems

This hierarchy reflects the progressive decoupling of biological and metallurgical functions, with each successive configuration offering greater independent control over cyanide generation and metal dissolution.

One-step bioleaching is typically the least effective configuration for SAC processing. Because untreated catalyst particles are present throughout microbial growth, high concentrations of dissolved metals, rapid cyanide consumption, and pH fluctuations inhibit bacterial activity and suppress cyanide production, particularly at higher pulp densities [8,20,44]. Similar limitations have been reported in e-waste bioleaching systems, where increasing solids loading decreases both microbial activity and metal recovery. Consequently, one-step bioleaching is primarily used for laboratory-scale investigations rather than industrial implementation.

The two-step configuration partially overcomes these limitations by separating microbial cultivation from waste exposure. Cyanogenic microorganisms are first grown to the logarithmic phase and maximum cyanide productivity before SAC is introduced, after which leaching proceeds [18]. This sequential approach reduces growth inhibition and improves recovery relative to one-step systems. A key residual limitation of the two-step approach is that active microorganisms remain present throughout leaching, so cyanide consumption and enzymatic degradation continue in parallel with metal dissolution, constraining lixiviant utilisation efficiency and suppressing Pt and Pd recoveries.

Spent-medium bioleaching addresses the limitations of the two-step approach by fully separating lixiviant generation from metal dissolution. Following microbial cultivation until the logarithmic phase, cells are removed by centrifugation or filtration, and the cyanide-rich supernatant is used for leaching under independently controlled conditions, typically completed within a single day. The absence of living cells eliminates cyanide degradation, allowing pH and dissolved oxygen to be optimised exclusively for metal dissolution rather than for microbial viability. Spent-medium systems have consistently achieved the highest recoveries among low-temperature batch configurations and currently represent the most mature biological approach for SAC treatment [9].

The highest overall recoveries have been reported for fully decoupled systems, in which biogenic cyanide production and metal dissolution operate as completely independent unit operations. This configuration eliminates all incompatibility between biological and metallurgical requirements, enabling each stage to be optimised in isolation. Two principal decoupled variants have been demonstrated for SAC treatment. In the high-temperature bio-cyanide configuration, Shin et al. [28] produced biogenic cyanide over 4–7 days, reaching concentrations of 954.8 mg L^−1^ in batch mode and 6594.5 mg L^−1^ under continuous operation, and subsequently applied a 1000 mg L^−1^ biogenic cyanide solution under pressurised leaching at 150 °C, achieving recoveries of 92.1% Pt, 99.5% Pd, and 96.5% Rh within 1–2 h, approaching those obtained with chemical NaCN leaching (100%, 99.9%, and 100%, respectively).

Despite their superior recovery performance, decoupled systems introduce greater process complexity. Requirements for pressurised reactors, high operating temperatures, specialised HCN trapping infrastructure, and ionic-liquid handling all increase capital and operating expenditure relative to simpler batch configurations. Also, high temperature and pressure conditions in bio-cyanide system raised important questions regarding the «green» classification of such systems. Although the lixiviant itself is biogenically produced, the energy requirements for pressurised heating reduce the environmental advantages relative to ambient-temperature operations. This highlights a fundamental tension within bioleaching research: the pursuit of maximum recovery through process intensification may partially compromise the sustainability benefits that originally motivate biological approaches.

Progressing toward industrial-scale implementation will therefore require careful balancing of recovery efficiency against process simplicity, energy consumption, reagent costs, and scalability.

**Figure 4:**
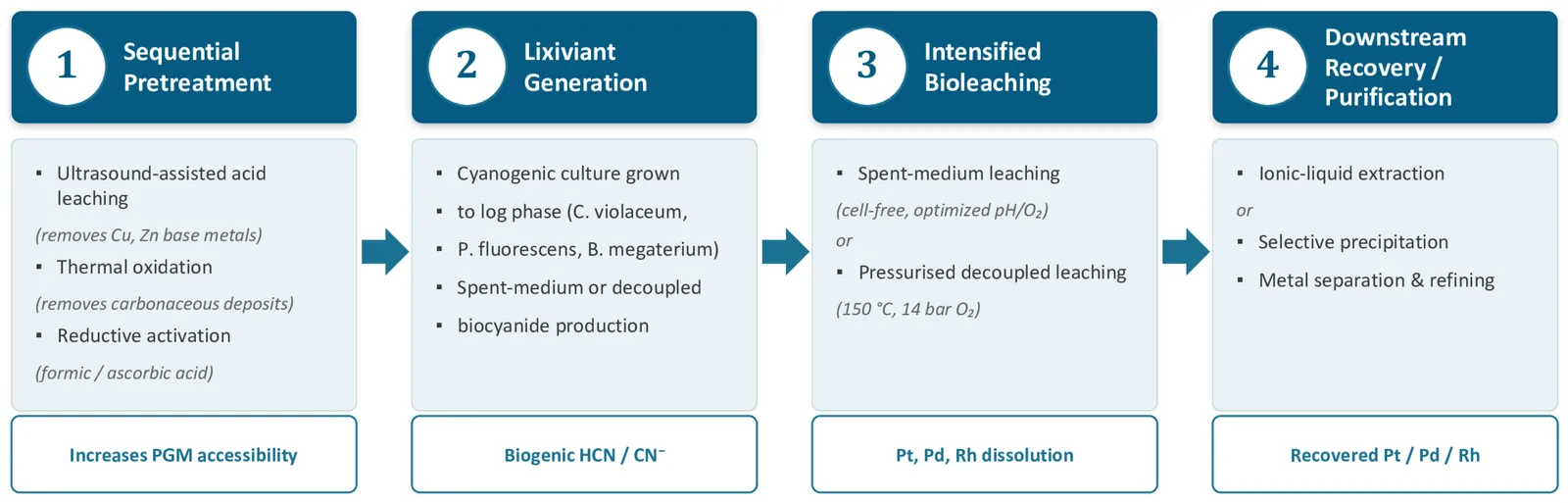
Integrated bio-hydrometallurgical flowsheet for PGM recovery from SAC.

An integrated process flowsheet combining these strategies is presented in Figure 4, illustrating the sequential stages of pretreatment, lixiviant generation, intensified bioleaching, and downstream purification.

## 7. Pulp Density and Metal Toxicity

Pulp density is one of the most important parameters, as it influences metal-ion concentrations, oxygen transfer, slurry rheology, microbial activity, and lixiviant availability. Studies involving *C. violaceum* and *Pseudomonas* species consistently show that increasing pulp density above approximately 1–2% (*w*/*v*) reduces metal recovery due to increased toxicity, oxygen limitation, and suppression of cyanide production [8,44].

This limitation is not unique to SAC processing; similar trends have been reported in metal bioleaching of electronic waste, where increased pulp density tends to reduce microbial growth and cyanide production, resulting in lower gold recovery [44]. Comparable effects have also been observed in bioleaching of spent batteries and refinery catalysts. In Zn-Mn battery bioleaching, increasing pulp density from 1 to 8% (*w*/*v*) reduced Zn and Mn recoveries from 100% and 94% to 29.9% and 2.5%, respectively [58]. Likewise, bioleaching of spent lithium-ion batteries showed substantial declines in Li and Co extraction as solids loading increased [59]. Collectively, these studies demonstrate that high pulp density represents a common constraint in bio-hydrometallurgical systems.

The underlying mechanism was investigated in detail during SAC bioleaching using *C. violaceum.* Intracellular reactive oxygen species (ROS), measured using DHR123, DCFH-DA and DHE fluorescent probes, increased sharply with increasing solids loading. At 0.5% (*w*/*v*), ROS levels remained relatively low and viable cell counts remained close to 108 CFU mL^−1^ throughout the bioleaching period. At 4% (*w*/*v*), ROS accumulation increased substantially, leading to a decline in viable cell counts from approximately 10^8^ to 10^2^ CFU mL^−1^ within three days. At pulp densities of 8–12% (*w*/*v*), viable cells disappeared entirely within 48 h. The progressive loss of cell viability directly suppressed cyanide production, with cyanide concentrations decreasing from 6.44 mg L^−1^ at 0.5% (*w*/*v*) to 0.80 mg L^−1^ at 4% (*w*/*v*), which resulted in significantly lower PGM recoveries. A mitigation strategy was developed by combining glutathione (GSH) and polyvinylpyrrolidone (PVP). GSH acted as an intracellular antioxidant that scavenged ROS, while PVP reduced bacterial attachment to catalyst particles and minimised localised metal toxicity. The addition of 0.6 g L^−1^ GSH and 0.4 g L^−1^ PVP reduced intracellular ROS levels by more than 60% at 4% (*w*/*v*) pulp density and maintained viable cell populations between 106 and 107 CFU mL^−1^ throughout the leaching period. Under these conditions, Pt, Pd, and Rh recoveries increased to 68%, 74%, and 86%, respectively, compared with approximately 30%, 33%, and 62% in untreated systems at the same pulp density [19].

The importance of antioxidant defence mechanisms is further supported by studies on acidophilic bioleaching microorganisms. Overexpression of glutathione synthetase in *A. ferrooxidans* increased intracellular glutathione concentrations and improved tolerance to otherwise inhibitory salt and pH conditions [60]. More broadly, microbial tolerance to high solids loading has been linked to a combination of extracellular protection mechanisms, including EPS production and biosorption, together with intracellular antioxidant pathways, efflux systems, and redox-buffering metabolites [19,40].

However, the literature offers limited direct guidance on what pulp density would need to be reached for cyanogenic PGM bioleaching specifically to approach economic competitiveness. Comparative evidence from related bio-hydrometallurgical systems, however, provides a useful benchmark. A techno-economic assessment of *A. ferrooxidans* based bioleaching for base-metal recovery (Cu from goethite) demonstrated financial viability at pulp densities up to 10% (*w*/*v*), with sensitivity analysis indicating that economic returns generally improve as pulp density increases, even where this comes at some cost to extraction efficiency [61]. Similarly, in a PGM-adjacent system developed by the same research group behind the α-alumina bioleaching pretreatment discussed in Section 4 [10], a biogenic thiosulfate–copper–ammonia leaching route achieved 93.2–96.2% Pd extraction from spent three-way catalysts at 5% (*w*/*v*) pulp density, using both biogenic and commercial thiosulfate sources [10]. This is substantially higher than the 0.5–1% (*w*/*v*) pulp densities typical of the cyanogenic bioleaching systems discussed throughout this review. Taken together, these findings suggest that pulp densities in the range of 5–10% (*w*/*v*) roughly five- to ten-fold higher than current cyanogenic PGM bioleaching systems achieve may represent a more realistic near-to-mid-term target for improving the economic viability of biological PGM recovery, rather than the 30–50% (*w*/*v*) densities used in conventional cyanidation. Reaching this intermediate range would likely require combining the oxidative-stress mitigation strategies discussed above (e.g., antioxidant supplementation, EPS-modulating agents) with adapted or engineered strains exhibiting greater tolerance to metal loading, since no cyanogenic PGM bioleaching study to date has demonstrated stable operation above ~4% (*w*/*v*) pulp density [19].

Overall, current evidence indicates that pulp density limitations arise primarily from metal-induced oxidative stress, which impairs microbial viability and lixiviant production. Future strategies for scale-up of PGM bioleaching will therefore require enhancing microbial tolerance to high-solids environments, whether through antioxidant supplementation, adaptive strain engineering, or process configurations that decouple biological and metallurgical stages.

## 8. Sustainability

The recovery of PGMs from SACs offers substantial environmental advantages over primary mining, as SACs contain significantly higher PGM concentrations than natural ores. As summarised in Table 5, production of 1 kg of Pt from primary resources typically requires processing approximately 150 tonnes of ore. It generates around 400 tonnes of waste rock and tailings, whereas recovery from SACs requires only about 2 tonnes of catalyst material. This substantial difference in resource intensity underpins the environmental benefits of secondary PGM recovery.

A life cycle assessment (LCA) comparing primary and recycled platinum (Pt) production revealed substantial environmental benefits associated with recycling. Resource consumption for primary Pt was 82.5 kg Sb-eq./kg, significantly higher than the 5.45 kg Sb-eq./kg for recycled Pt, representing an environmental reduction effect of approximately 93%. In terms of greenhouse gas emissions, primary Pt generated 1.35 × 10^4^ kg CO_2_-eq./kg, whereas recycled Pt emitted only 6.94 × 10^2^ kg CO_2_-eq./kg, corresponding to a reduction of approximately 95% [62]. Similar trends are observed in energy demand and water consumption, largely because recycling eliminates the energy-intensive processes of mining, comminution, flotation, smelting, and refining.

Within secondary recovery technologies, bioleaching offers additional sustainability benefits because lixiviants are generated biologically under relatively mild operating conditions. Atom economy (AE) values for the cyanidation reactions of PGM ranged from 80 to 90%, indicating that almost all atoms in the reactants (cyanide, oxygen, water) end up in the desired metal-cyanide complex. Effective mass yield, which measures product mass relative to non-benign reagents, followed the same trend as percentage yield, with *C. violaceum* giving the highest overall value (~75.1%) due to near-complete rhodium extraction [15]. Percentage yield varied widely (28–91% for Pt, 32–95% for Pd, 62–100% for Rh) depending on pulp density, bacterial strain, and pretreatment. The highest reported yields (91% Pt, 95% Pd, 100% Rh) were achieved with *C. violaceum* using formic acid pretreatment and spent-medium leaching [8,15].

Waste metrics revealed both the strength and the weakness of biological cyanidation. The simple E-factor (EF) excluding water was exceptionally low (0.35–0.53 wt.%. ratio of waste to product), which reflects the catalytic, in situ nature of biogenic cyanide and the absence of large inorganic salt waste streams. For comparison, conventional hydrometallurgical processes have simple E-factors of 80–300 kg/kg of waste to product, and pharmaceutical manufacturing ranges from 25 to 100 kg/kg [15,63,64]. Bioleaching thus compares favourably with bulk chemical production. However, the complete E-factor (cEF), which includes water, was dramatically higher ((0.5% *w*/*v*), 222.70% for *P. fluorescens* and 235.84% for *B. megaterium*) and was dominated by the water used in dilute microbial cultivation. The process mass intensity (PMI_2_), defined as total mass inputs divided by product mass, followed the same trend (approximately 16,500–19,300 kg/kg), confirming that the vast majority of input mass (water) does not report to the product [16].

Along with these metrics, the core sustainability bottleneck of current cyanogenic bioleaching was identified not as reagent toxicity or energy intensity, but as water intensity and the low pulp densities required to maintain bacterial viability (0.5–1 wt.%) [19]. These values are 30–50 times lower than those used in industrial chemical cyanidation (30–50 wt.%) [16,32]. The high intrinsic value of PGMs further enhances the sustainability case for biological recovery. Beyond metal production, bioleaching systems may facilitate the generation of value-added products such as biogenic Pt and Pd nanoparticles, which possess catalytic and antimicrobial properties. Such opportunities could improve overall process economics while supporting circular-economy objectives. Overall, available life-cycle evidence indicates that recovery of PGMs from SACs offers major environmental advantages over primary mining, including substantial reductions in waste geneation, greenhouse gas emissions, energy consumption, and resource use.

## 9. Conclusions

This review explored the development of bio-based approaches for recovering PGMs from SACs and highlighted the transition from early proof-of-concept studies to increasingly effective and sustainable recovery strategies. The findings demonstrated that successful PGM bioleaching requires the integration of catalyst pretreatment, microbial lixiviant production, and process engineering rather than optimisation of any individual component alone. Cyanide bioleaching remains the most effective biological route for direct PGM dissolution, as cyanide forms highly stable complexes with PGMs under alkaline conditions.

The pretreatment strategies of thermal oxidation, acid washing, reductive activation, and matrix conditioning improve catalyst accessibility by removing the carbonaceous deposits, dissolving the competing metals, reducing the surface passivation, and exposing the encapsulated PGMs. The acidophilic chemolithotrophs contribute further through the matrix degradation and the base-metal removal, while the cyanogenic microorganisms provide the lixiviants required for direct dissolution of PGMs. These complementary functions support the development of hybrid bio-hydrometallurgical flowsheets.

The kinetic analyses indicate that PGM bioleaching is governed by coupled biological, chemical, and transport phenomena. Although the initial dissolution may be reaction-controlled, the diffusion through porous catalyst structures increasingly dominates at higher conversion levels. Future modelling efforts should therefore integrate factors such as the microbial growth, the lixiviant generation, the interfacial reactions, and the intra-particle mass transfer within unified multi-scale frameworks capable of supporting reactor design and scale-up.

From a sustainability perspective, the recovery of PGMs from SACs offers opportunities along with environmental advantages over primary mining, including reductions in waste generation, greenhouse gas emissions, energy consumption, and material throughput. Bioleaching further contributes through reagent consumption, favourable atom efficiency, and operation under comparatively mild conditions. Several research gaps remain before large-scale deployment can be achieved, including addressing the metabolic engineering of cyanogenic microorganisms to improve cyanide productivity and metal tolerance, developing high-solids bioleaching systems, validating integrated kinetic models, and conducting pilot-scale techno-economic assessments under realistic operating conditions. Further investigation into the valorisation of bioleach residues and biologically synthesised PGM nanoparticles may also provide opportunities to enhance process economics and support circular-economy objectives.

Overall, current evidence suggests that bioleaching is unlikely to replace conventional hydrometallurgical processing as a standalone technology in the near future. Instead, its greatest potential lies within integrated bio-hydrometallurgical flowsheets that combine biological selectivity with established metallurgical recovery technologies, offering a more sustainable pathway for green recovery of critical PGMs from secondary resources.

## Figures and Tables

**Table 1:** Characteristics of pyrometallurgical, hydrometallurgical, and biohydrometallurgical methods for PGM recovery from SACs.

Method	Conditions	PGM Recovery	Energy Demand	Advantages	Disadvantages	References
1. Pyrometallurgy Smelting, roasting, plasma treatment	1200–1600 °C Large-scale electric arc or plasma furnaces; air/O_2_ atmosphere	80–99% Pt, Pd, Rh simultaneously	Very high, 1400–3400 MJ per kg Pt dominates operating cost	- Industrially mature; large throughput - Processes mixed/heterogeneous feeds - Short cycle time - All PGMs recovered in a single step	- Extreme energy demand - High CO_2_ emissions: 41.35 kg CO_2_ per kg Pt from ore (4× higher than SAC recycling) - High SO_2_ emissions: 7267 kg SO_2_ per kg Pt from ore - Costly off-gas scrubbing required - Volatile PGM losses (esp. Rh) - High capital and operating costs - No selectivity leading to downstream refining	[2,5,7,11]
2. Hydrometallurgy Acid/alkaline leaching, solvent extraction, precipitation, electrodeposition	20–200 °C Aqua regia, HCl/Cl_2_, NaCN or KCN salts; atmospheric to mild pressure	85–99 High selectivity achievable via solvent extraction	Moderate-high Lower than pyro; significant reagent manufacture energy	- Lower temperature vs. pyrometallurgy - High selectivity individual PGMs separable - Scalable to smaller operations - Established industrial flowsheets	- Toxic reagents (aqua regia, HCN gas, cyanide salts) - Large acidic/cyanide wastewater volumes - Corrosion of equipment - Complex multi-stage purification - Secondary pollution risk; high disposal cost - Requires pre-dissolution/comminution step	[2,5,7,12]
3. Biohydrometallurgy Cyanogenic bioleaching; acidophilic chemolithotrophs; microbial-assisted leaching	20–80 °C pH 9–11 (cyanogenic) or pH 1–3 (acidophilic); ambient pressure; biogenic lixiviants	- 10–100% Highly variable; up to ~100% Rh, 95% Pd, 91% Pt with optimal pretreatment	Low Ambient conditions; no smelting energy; dominated by water use (high PMI)	- Low energy consumption - Mild operating conditions - Reduced toxic chemical use vs. hydro - Lower GHG emissions - Selective recovery potential - Suitable for low-grade/complex feeds - Simple E-factor 0.0035–0.005 kg/kg	- Slow kinetics of days to weeks - Sensitive to PGM toxicity and pH - Low pulp density (0.5–1 wt.%) leading to high PMI - Recovery variable; strain- and pretreatment-dependent - Scale-up challenges; pre-industrial TRL - Requires controlled bioreactors - HCN volatilisation risk at below pH 9	[7,13,14,15,16]

**Table 2:** Effect of sequential pretreatment methods on PGM leaching rates.

Step	Objective	Pt Recovery (%)	Pd Recovery (%)	Rh Recovery (%)	Microorganism	References
1. Ultrasound-assisted nitric acid	Remove base metals (Cu, Zn, Fe)	38	44	91	*Pseudomonas* *fluorescens*	[18]
		35	41	82	*Bacillus megaterium*	
2. Sequential pretreatment (Heat oxidation 850 °C + Sonication + Formic acid reduction)	Remove base metals (Cu/Zn)	91	95	100	*Chromobacterium* *violaceum*	[8]
3. Bioleaching + aqua regia	Remove REEs; surface activation	100	100	Not reported	*Aspergillus* *niger* ZRS14	[26]
4. Bioleaching (pre-treatment)	Dissolve the alumina washcoat layer	Not reported	Not reported	Not reported	*Acidithiobacillus* *thiooxidans*	[10]
5. Bioleaching (oxalic acid-rich spent medium)	Biorecovery of metals and enrichment of REEs	60.9	73.7	Not reported	*Aspergillus niger*	[27]
6. Accumulation + Pressure leaching	Decouple biological growth from harsh leaching conditions	92.1	99.5	96.5	*Chromobacterium* *violaceum*	[28]
7. Ultrasound-assisted	Removal of soot & increased surface area	27.3	8.0	6.6	*Microbacterium* sp. *T2* *Microbacterium* sp. *AG*	[29]

**Table 3:** Acidophilic bioleaching across different substrate types and mechanisms supporting acidophilic bioleaching as a matrix-conditioning pretreatment for SACs.

Substrate Type	Key Microorganisms	Mechanism	Outcome	Reference
Metallic substrate converter FeCrAl metallic carrier PGM-coated	*A. ferrooxidans* + *A. thiooxidans* (mixed culture)	Fe^3+^ mediated oxidation destabilises FeCrAl matrix under strongly acidic conditions	Metallic carrier weakened; biological Fe dissolution exceeds abiotic controls; downstream PGM liberation improved	[37]
Iron ore concentrate Sulfide-bearing	*A. thiooxidans*	Sulfur oxidation produces H_2_SO_4_; green biodesulfurisation removes sulfide phases	Effective sulfide removal; improved ore purity	[38]
Contaminated soil Toxic metal(loid)-bearing matrix	*Acidithiobacillus* dominated consortia	Acid + Fe^3+^ attack dissolves metal-bearing mineral phases; mobilises toxic metals.	Effective extraction of toxic metal(loid)s; mechanisms characterised	[39]
Refractory sulfide ore Industrial bioleaching	*A. ferrooxidans*	Genome-informed sulfur and iron oxidation pathways;	Metabolic pathways characterised; benchmark organism for acidic bioleaching	[35]
Various metal-bearing substrates Broad acidophilic bioleaching	*Acidithiobacillus* spp. and related acidophiles	H_2_SO_4_ + Fe^3+^ attack on silicate and sulfide matrices; biotechnological characteristics reviewed	Broad metal extraction; base metal removal reduces downstream competition	[12]
Sulfide/mixed mineral substrates Potential relevance to refractory PGMs	*A. ferrooxidans* + *A. thiooxidans* (synergistic consortium)	Synergistic Fe and S oxidation; combined acid + oxidant generation accelerates matrix dissolution	Enhanced dissolution efficiency; encapsulating phases broken down	[40]
α-Alumina layer of spent three-way catalysts	*A. thiooxidans*	Acid bioleaching of Al by biogenic sulfuric acid; bacteria-free biogenic acid vs. stationary-phase bacteria vs. commercial H_2_SO_4_	Max. Al leaching 54.5% at three-way catalyst (TWC) pulp density 5% *w*/*v* using biogenic acids with stationary-phase bacteria; superior to commercial H_2_SO_4_ (24.7%) and bacteria-free biogenic acid (23.4%)	[10]
Spent automotive catalysts (focus on magnetic separation and base metal removal)	Acidophilic bacteria	Bioleaching for base metal removal; magnetic separation as complementary step	Base metals (Fe, Cr, Al) were solubilised; magnetic separation led to conflicting performance in PGM recovery by removing magnetic base-metal particles.	[17]

**Table 4:** Cyanide affinities and competitive complexation behaviours of metals in spent automobile catalysts.

Metal	Role	Dominant Cyano-Complex	log β (Stability)	CN^−^ Affinity	Competitive Effect on PGMs	References
Pt	Catalyst	[Pt(CN)_4_]^2−^	~40–41	Very high	Blocked by competing base metals	[45]
Pd	Catalyst	[Pd(CN)_4_]^2−^	62.3	Exceptionally high	Strongly competes with base metals; extreme thermodynamic preference for CN^−^	[46]
Ni	Substrate alloy	[Ni(CN)_4_]^2−^	30.5	High	Strong competition; consumes 4 CN^−^ per Ni	[45]
Rh	Catalyst	[Rh(CN)_6_]^3−^	~47	High	Slower dissolution; requires free CN^−^	[47]
Cu(I)	Contaminant	[Cu(CN)_3_]^2−^	~27	High	Consumes 3 CN^−^ per Cu^2+^; major competitor	[25]
Zn	Minor contaminant	[Zn(CN)_4_]^2−^	~19	Moderate	Moderate consumption (4 CN^−^ per Zn); complex weakly stable	[24]
Fe(II)	Dissolved iron from Fe-bearing phases	[Fe(CN)_6_]^4−^	31.8	High	Oxidation to Fe(III) followed by hydrolysis produces ferric hydroxide precipitates; consumes 6 CN^−^ per Fe	[48]
Fe(III)	Oxidised surface	[Fe(CN)_6_]^3−^	~39	Extremely high	Ferricyanide formation; severe CN^−^ loss	[48]

**Table 5:** Comparative Environmental Metrics: Primary PGM Production vs. Secondary Recovery from SACs.

Parameter	Primary Production (per kg Pt)	Secondary Recovery (per kg Pt)	Reduction	References
Ore/waste processed	150 Mg ore	2 Mg SAC	~98%	[7]
GHG emissions	~13,500 kg CO_2_-eq	~694 kg CO_2_-eq	~95%	[62]
Water consumption	100–1200 m^3^/kg PGM	3–6 evalum^3^/kg PGM	Significant reduction	[2]
Energy consumption	18.9–254.9 GJ/kg PGM	1.4–3.4 GJ/kg PGM	-	[2]
Solid waste generation	~400 Mg/kg Pt	Not reported	-	[7]
Chemical consumption	High (acids, cyanide)	Low (biogenic cyanide)	~90% (estimated)	[15]

## Data Availability

No new data were created or analyzed in this study. Data sharing is not applicable to this article.
